# Injection laryngoplasty during transoral laser microsurgery for early glottic cancer: a randomized controlled trial

**DOI:** 10.1186/s40463-022-00564-y

**Published:** 2022-03-22

**Authors:** Ayham Al Afif, Matthew H. Rigby, Colin MacKay, Timothy F. Brown, Timothy J. Phillips, Usman Khan, Jonathan R. B. Trites, Martin Corsten, S. Mark Taylor

**Affiliations:** 1 Queen Elizabeth II Health Sciences Centre, 3rd Floor Dickson Building, 5820 University Avenue, Halifax, NS B3H 1Y9 Canada; 2 Department of Surgery, Queen’s University, Victory 3, Kingston General Hospital, 76 Stuart Street, Kingston, ON K7L 2V7 Canada; 3 University of Alabama at Birmingham, 1155 Faculty Office Tower, 510 20th Street South, Birmingham, AL 35233 USA

**Keywords:** Early glottic cancer, Transoral surgery, Laryngoplasty, Voice outcomes

## Abstract

**Background:**

Transoral laser microsurgery is widely used for treating T1/T2 glottic cancers. Hyaluronic acid (HA) is commonly used in vocal cord augmentation. We investigated the impact of intra-operative injection laryngoplasty on voice outcomes in early glottic cancer.

**Methods:**

Twenty patients were randomized to the treatment group receiving HA injection to the vocal cord contralateral to the lesion; or the control group, receiving no injection. Patients had a Voice Handicap Index-10 (VHI-10) questionnaire and a Maximum Phonation Time (MPT) measurement preoperatively and at 3, 12 and 24 months post-operatively. Mean change in VHI-10 and MPT, compared to baseline and between time points, were compared. Survival estimates were calculated.

**Results:**

Mean VHI-10 scores improved over time amongst all patients. There were no changes in mean VHI-10 from pre-operative values to 3, 12 or 24 months post-operatively. There were no significant differences when comparing various timepoints between groups. There were no significant changes in MPT amongst the groups, or the time-points compared. Two-year overall survival was 91.7%; disease free survival was 80.9%; no difference in recurrence free survival was seen between the groups.

**Conclusion:**

Subjective voice scores improved over time in both groups; there were no improvements in VHI-10 or MPT scores in the injection group, over control, at any time points. We saw no significant impact for intra-operative HA injection laryngoplasty on subjective or objective voice outcomes following surgery for early glottic cancers.

**Graphical abstract:**

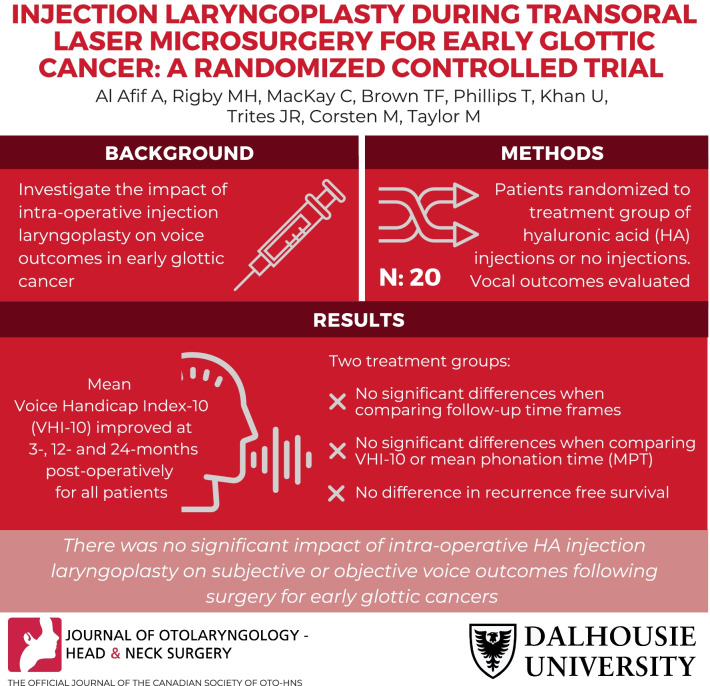

## Introduction

Laryngeal squamous cell carcinoma (SCC) is the commonest malignancy of the head and neck [1]. The incidence of laryngeal SCC has been slowly decreasing owing to the declining number of smokers in the population. The glottis accounts for 60% or more of all cases. Over 50% of patients present with early glottic lesions (T1/T2, N0) [2, 3]. This is due partly to the prompt onset of symptoms which can expedite diagnosis and management. Early glottic cancers have a favorable overall survival (OS) of 83% at 5 years [3].

Early glottic cancers can be managed with radiation therapy (RT) or surgery. Over recent years, transoral laser microsurgery (TLM) has been favored over traditional open approaches. First described in 1972 by Strong and Jako [4], TLM is now the standard of care in many centers [5]. It offers several advantages over RT; it avoids indirect damage to adjacent tissue, it is provided in a single setting where re-operation is possible in a timely fashion, and also allows for adequate margin assessment [6]. Numerous factors, such as age, ongoing smoking, patient expectation and vocal use can impact voice outcomes after TLM [7]. The extent of the resection also plays a crucial role in voice quality post-operatively. The European Laryngological Society (ELS) proposed a classification system to describe the extent of endoscopic cordectomies [8]. Deeper and more extensive cordectomies (type III-V) result in worse voice outcomes compared to more superficial cordectomies (type I and II) [9].

Several studies have demonstrated comparable oncologic outcomes between the modalities in treating T1 and T2 lesions [10, 11]. However, there remains much debate as to what tool offers more superior voice outcomes. Interpreting post-treatment voice outcomes in early laryngeal cancer can be challenging. Objective voice outcomes include maximal phonation time (MPT) and changes in fundamental frequency (jitter), amplitude (shimmer) or noise-to-harmonic ratio. Commonly used subjective outcome scales include the Voice Handicap Index (VHI), and its abbreviated form the VHI-10 scale [12]. Many studies have demonstrated a higher laryngeal preservation rate for TLM in treating early glottic cancers when compared to RT, ranging from 93 to 100% [10, 11, 13, 14]. A systematic review by Guimaraes et al*.* demonstrated better subjective and objective voice outcomes in patients with T1a lesions after RT compared to TLM [15]. However, a review by Greulich et al*.* demonstrated no difference in voice outcomes between the modalities in T1 lesions [16]. The only RCT comparing the two modalities demonstrated similar subjective voice outcomes in T1 lesions. However, the TLM group was found to have a breathier voice and a larger glottic gap [17].

Injection thyroplasty is widely used in the management of vocal cord paralysis. Closure of the glottic gap is thought to improve voice quality and strength, reduce breathiness and ameliorate the risk of aspiration [18]. Hyaluronic acid (HA) is a the most widely used injectable, with a predictable and well-studied safety profile [19]. When TLM is used to treat early glottic cancers, the size of the glottic gap is influenced by the amount of tissue resected for oncologic control. The degree of post-operative dysphonia correlates directly with the size of the glottic gap [20].

The aim of this single-blinded RCT was to investigate the impact of intra-operative injection laryngoplasty, to the vocal fold contralateral to the lesion, on voice outcomes in patients undergoing TLM in our institution. We hypothesized that injecting the contralateral vocal fold will improve voice outcomes post-TLM by minimizing the glottic gap.

## Materials and methods

### Study design

Ethics approval was obtained from the Nova Scotia Health Authority Research Ethics Board (#1020322). We included males and females, 18 years of age or older with a biopsy-proven T1a, T1b or T2 glottic SCC amenable to CO_2_ TLM resection. A total of 39 patients were included in our study. The following exclusion criteria were applied:

- Previous radiotherapy to the head and neck.
- Palpable, or radiographic, pathological lymphadenopathy.
- Allergy, or sensitivity, to HA or components of the injectable.
- Neurological disorder affecting phonation, such as multiple sclerosis or stroke.

Patients were randomized to the treatment group or the control group in a 1:1 ratio. Randomization was achieved through a computer generated string. The treatment group received HA injection to the vocal cord contralateral to the lesion during the TLM resection. In patients with T1b lesions, the vocal cord with less disease burden was received the injection. Patients in the control group received no injection during the procedure. All resections and injections were performed by the senior author (S.M.T) to maintain uniformity. Blinding was strictly maintained amongst all patients throughout the trial. Informed consent was obtained during the initial clinic visit. By consenting, the patients were informed of their involvement in the trial, but not whether they would undergo injection laryngoplasty. Subsequently, the receipt of injection laryngoplasty, or lack thereof, was not disclosed until after the last VHI-10 and MPT measurements were obtained in clinic.

**Fig. 1:**
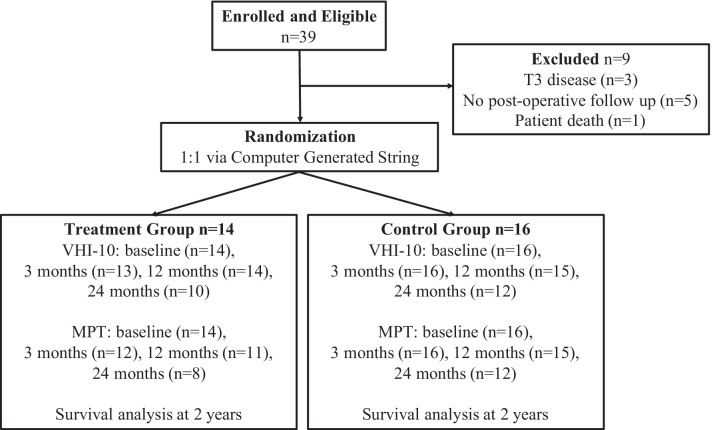
Study design and randomization as per Consolidated Standards for Reporting of Trials (CONSORT) statement guidelines

The primary outcome was subjective and objective voice measures, through VHI-10 scores and MPT respectively. Secondary outcomes were overall and recurrence free survival. Pre-operatively, and at 3, 12 and 24 months post-operatively, all patients filled out a VHI-10 questionnaire and underwent an MPT measurement. MPT was measured in seconds as the longest sustainable vowel sound after a deep breath. MPT value recorded was the average of 3 attempts. Demographic data, survival as well as recurrence were recorded throughout the trial. Patients diagnosed with a T3 lesion intraoperatively were excluded from further primary outcome analysis. Similarly, patients who developed a recurrence throughout the trial necessitating further surgery were excluded from further analysis. Lastly, we also excluded patients with no post-operative VHI-10 and/or MPT score recorded (Fig. 1).

### Surgical procedure

All procedures were done under general anesthetic. Patients were intubated with a laser-safe endotracheal tube. Microlaryngoscopy and suspension were then performed, and the operating microscope was adjusted to a focal distance of 400 nm. The CO_2_ laser was set to 1–2 Watts ultra-pulse and the micromanipulator was mounted. The tumor was split to identify the depth of infiltration. The posterior portion of the tumor was resected first, followed by the anterior portion, with a 1–2 mm margin. Occasionally, the false cord was partially resected for improved visualization. Following tumor resection, the appropriate margins were sampled and sent for intra-operative frozen section analysis. Hemostasis was maintained with topical adrenaline (1:100,000). Between 0.2–0.6 mL of Perlane® (Gladerma Inc., Thornhill, Ontario) was injected using a long Sataloff needle (Integra Lifesciences, Burlington, Ontario) into the vocal fold immediately lateral to the vocal ligament and vocal process until adequate medialization was achieved. Medialization was deemed adequate when vocal process of the affected cord approached midline. The patient was then extubated, reversed and transferred to the post-anesthesia care unit. No post-operative voice rest was required. All patients were placed on a proton pump inhibitor therapy for the first 3 months post-operatively (Fig. 1).

### Statistical analysis

A power calculation was performed prior to commencing the trial. Baseline estimates of VHI-10 scores for patients with T1 and T2 glottic cancers were based on the values reported by Kerr et al. [21]. We considered a change in VHI-10 of 4 points (10%) to be significant [22]. As such, we determined that the minimum number of patients per arm is 20. Pearson’s Chi-squared, Mann–Whitney U, and Fisher’s Exact tests were used to determine the comparability of the demographics of the treatment and control groups. Mean changes in VHI-10 scores were compared between the groups using independent t-tests. Paired t-tests were used to compare the mean VHI-10 scores between each time point. Survival estimates were calculated using Kaplan–Meier survival curves. For this study, disease free survival (DFS) is defined as time to recurrence or second primary. All statistics were performed in the Statistical Package for Social Sciences (SPSS, Version 24, SPSS Incorporated, Chicago, IL).

## Results

### Group demographics

A total of 39 patients were recruited to the trial. Three were found to have T3 disease intraoperatively, and 3 required additional surgery during the trial and hence were excluded from primary outcome analysis. Three patients had no follow-up data post-operatively and were similarly excluded; one patient died unexpectedly; 2 patients elected not to return for any post-operative follow-up, and were subsequently referred to another surgeon.

A total of 30 patients were included in the study, 27 males and 3 females. There were 14 patients in the treatment group and 16 in the control group. The average age of our patients was 70.2 years with a standard error (SE) of 1.6 years. There were no differences between the groups with regards to sex, T-stage, smoking or alcohol history (Table 1).

**Table 1:** Patient demographics

	Overall (n = 30)	Treatment (n = 14)	Control (n = 16)	p-value
*Mean age (SE)*	70.2 (1.6)	69.8 (2.3)	70.5 (2.2)	0.790†
*Gender (%)*				0.586‡
Male	27 (90)	12 (86)	15 (94)	
Female	3 (10)	2 (14)	1 (6)	
*T stage (%)*				
T1a	4 (13)	2 (14)	2 (13)	
T1b	9 (30)	3 (21)	6 (38)	0.685
T2	17 (57)	9 (64)	8 (50)	
*Smoking history (%)*				
None	1 (3)	1 (7)	0	
Ex-smoker	22 (73)	10 (71)	12 (75)	0.550
Smoker	7 (23)	3 (21)	4 (25)	
*Alcohol history (%)*				
None	19 (63)	8 (57)	11 (69)	
Ex-user	6 (20)	3 (21)	3 (19)	0.762
User	5 (17)	3 (21)	2 (13)	

Differences were determined using Pearson Chi-squared tests except for mean age which was determined by Mann–Whitney U test (†) and gender which was determined by Fisher’s Exact Test (‡)

We also analyzed the extent of surgical resection. As per the European Laryngological Society (ELS) Cordectomy Classification [8]. The most common cordectomy type was V amongst both groups, performed in 60% of the treatment group and 75% of the control group. There was no significant difference between the two groups with regards to cordectomy type or anterior commissure resection (Table 2).

**Table 2:** Cordectomy type and anterior commissure resection amongst the groups

	Treatment (n = 14)	Control (n = 16)	p-value
*ELS*^*†*^* cordectomy type (%)*			0.3252
I	0 (0)	2 (12)	
II	2 (14)	1 (6)	
III	1 (7)	1 (6)	
IV	2 (14)	0 (0)	
V	9 (60)	12 (75)	
*Anterior commissure resection (%)*			0.122^‡^
Resected	7 (50)	13 (80)	
Spared	7 (50)	3 (20)	

Pearson Chi-square test was used for comparison of ELS Cordectomy type

^‡^Fisher’s exact test was used to compare anterior commissure resection

^†^ELS: European Laryngological Society

### Voice outcomes

Compared to mean pre-operative VHI-10 scores, the whole cohort saw a significant improvement at 3, 12, and 24 months post-operatively. There were no significant changes in VHI-10 when individual time points were compared. There were no significant differences in mean MPT between pre- and post-operative time points, or when any post-operative time points were compared (Table 3).

**Table 3:** Comparison of mean VHI-10 and MPT performance for all patients at various time points

Group	VHI-10			MPT		
	N	Mean (SD^†^)	p-value	N	Mean (SD)	p-value
Pre-Op^‡^	29	16.8 (10.1)	0.014*	28	11.53 (4.7)	0.371
3 months		12.1 (10.9)			10.73 (5.6)	
Pre-Op	26	16.0 (9.8)	0.010**	26	11.86 (4.3)	0.289
12 months		11.5 (10.4)			13.97 (9.8)	
Pre-Op	21	16.7 (10.1)	0.002**	21	11.52 (4.3)	0.700
24 months		9.0 (7.9)			12.13 (7.2)	
3 months	25	11.8 (11.2)	0.951	24	11.18 (5.3)	0.104
12 months		11.9 (10.4)			14.01 (10.3)	
3 months	20	11.4 (10.9)	0.329	19	10.80 (5.7)	0.320
24 months		9.4 (7.9)			12.38 (7.5)	
12 months	21	11.7 (10.5)	0.084	21	13.79 (10.8)	0.180
24 months		9.0 (7.9)			12.13 (7.2)	

N: number of patients in each t-test comparison. Paired t-test were used.

*p-value < 0.05, **p-value < 0.01

^†^SD: standard deviation

^‡^Pre-op: pre-operatively

**Fig. 2:**
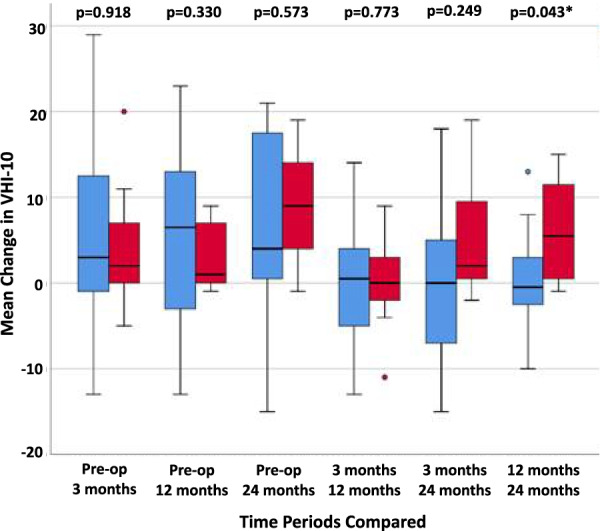
Box plot for mean change in VHI-10 scores. Red: treatment; blue: control. Pre-op: pre-operative. *p < 0.05

When comparing VHI-10 scores amongst the treatment groups, there were no significant differences in mean scores between pre-operative values to 3, 12 or 24 months post-operatively. When comparing mean VHI-10 scores between 12 and 24 months, the treatment group showed a greater improvement when compared to the treatment group (p = 0.043; Fig. 2).

**Fig. 3:**
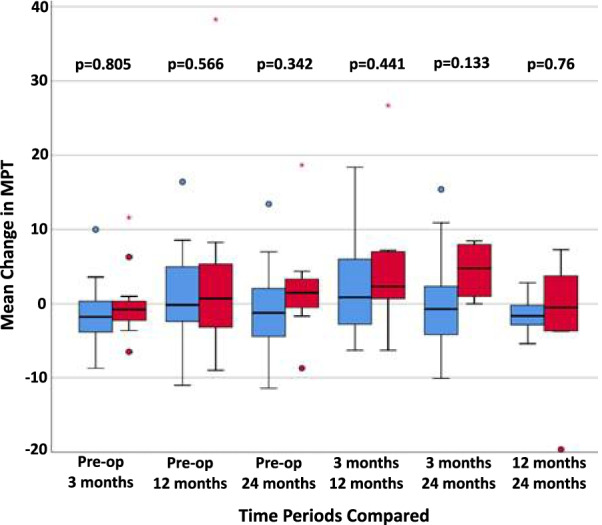
Box plot for mean change in MPT scores. Red: treatment; blue: control. Pre-op: pre-operative

There were no significant differences between the groups in mean MPT scores when comparing pre-operative values to 3, 12 or 24 months post-operatively, or when comparing individual time points (Fig. 3).

We also analyzed VHI-10 and MPT scores for T1 and T2 lesions separately, and found no significant differences depending on T-stage, over time. Similarly, there were no significant differences in VHI-10 or MPT scores in patients over the age of 70 years, compared to those younger.

### Survival analysis

**Fig. 4:**
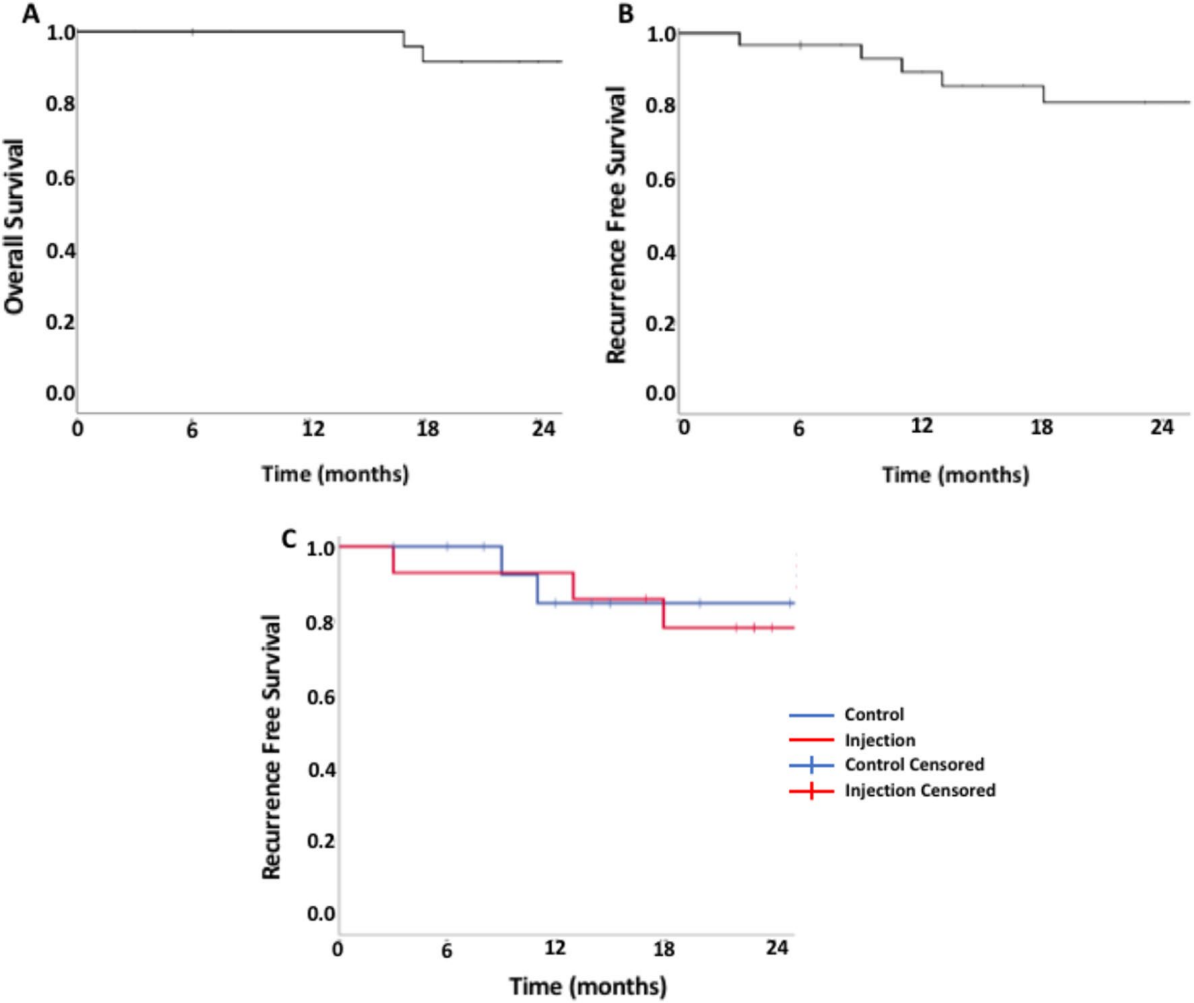
Overall (**A**) and recurrence-free (**B**) survival in all patients; recurrence free-survival (**C**) between the groups

Kaplan–Meier analysis showed an overall survival (OS) for the cohort of 91.3%, SE 5.9% at 2 years (Fig. 4A). DFS was 80.1%, SE of 8.1% at 2 years (Fig. 4B). There were no significant differences in recurrence free survival (RFS) at 2 years between the treatment and control groups; 84.6% SE 10% and 77.9% SE 11.3% respectively (p = 0.941, Fig. 4C). Two patients required a total laryngectomy for salvage, one in each treatment group, for a laryngeal preservation rate of 93.3% for the cohort.

## Discussion

There is not a gold standard tool to quantify voice outcomes after surgery for early glottic cancers. In fact, a myriad of subjective and objective post-operative voice assessment tools have been reported in the literature, and at various time points post-operatively. Furthermore, there is not a single objective parameter that can be used to prognosticate a patient’s subjective evaluation of their own voice [23]. Objective measures of voice have been suggested to have poor validity and reproducibility [24]. This makes interpreting post-operative voice outcomes rather challenging [25].

This study reports on the outcomes of our single-blinded randomized controlled trial investigating the impact of intra-operative injection layrngoplasty on voice outcomes in early glottic cancer. Our whole cohort showed an improvement in subjective voice outcomes over time. We found no significant differences in VHI-10 scores between baseline and 3, 12 or 24 months in any group. When comparing individual time points, we also found no significant improvement in VHI-10 scores. Although the mean VHI-10 change between 12 and 24 months favored the treatment group (p = 0.043), this is unlikely to be a statistically significant change, and is probably related to multiple comparisons. Overall, there were clearly no differences in voice outcomes amongst the groups. As such, we did not feel that any additional sensitivity analyses would alter our findings or conclusions. Much debate remains regarding the optimal time point to assess voice quality after TLM. Lane et al*.* found that VHI-10 scores show maximal improvement 5.5 months after TLM [26]. Other authors argue that voice assessments should be performed at least 6–12 months post-TLM to allow post-operative edema to resolve [7]. It is therefore possible that any differences in VHI-10 may have not been captured at time points recorded in our study.

MPT is a reliable and reproducible objective measure of glottic function [27]. We detected no differences in MPT between the groups at any time points, or compared to baseline. Unlike VHI-10 scores, the cohort saw no improvement in MPT scores over time. MPT is influenced by several factors including sex, age and lung elasticity [28]. The lack of improvement in MPT may be explained by the propensity for advanced cordectomies in our cohort, which can result in worse glottic competence and MPT scores.

Several factors influence voice outcomes after TLM. These include, age, voice use and patient expectations [7]. A standardized classification system to describe the extent of endoscopic cordectomies was proposed by the ELS [8]. Haddad et al*.*, demonstrated that patients who underwent ELS type I or II cordectomies had complete glottic closure patterns, and relied less on supraglottic vibratory mechanisms. In contrast, type III and IV cordectomies commonly resulted in incomplete closure patterns and mucosal waves, with a propensity for supraglottic and ventricular compensatory mechanisms [29]. It has also been shown that the pattern of supraglottic compensation is dictated by the extent of glottic resection. With larger glottic gaps, more supraglottic hypertrophy and arytenoid hyperadduction were observed [30]. We therefore hypothesized that injecting the vocal fold contralateral to the lesion intra-operatively will improve voice quality by improving glottic closure, hence reducing the development of supraglottic and ventricular compensatory voicing mechanisms, and enhancing voice quality. Injecting the cord contralateral to the lesion avoids introducing the injectable into the operated field, as the oncologic impact of HA injection into a tumor resection bed is unknown. This also avoids the spillage of the injectable. Additionally, injecting the operated cord may not always be feasible when the thyroarytenoid muscle has been resected.

A systematic review by Bertelsen et al. found that medialization thyroplasty is a useful tool in enhancing voice after cordectomy [20]. Villaret and colleagues reported on voice outcomes following intra-operative autologous fat injections into the neocord after type III laser cordectomy. Up to 8 cc of fat was used per patient. An improvement in subjective, but not objective voice outcomes were observed 1 year after surgery [31]. Guven et al. found that autologous fat injections into the neocord after cordectomy improved the glottic gap improved, but resulted in a deterioration of the mucosal wave [32]. Volume over-injection by 50% is advocated when using fat to counteract reabsorption [33]. In most studies, augmentation procedures are performed 6–12 months post-operatively. In our study, we performed the laryngoplasty intra-operatively. Our study is also the first to inject the vocal fold contralateral to the lesion.

Many injectables have been used in thyroplasty [34]. HA has been shown to improve glottic function in patients with vocal cord paralysis [19]. Although the injectable only lasts for 3 months, its clinical effect can last for up to 12 months [34]. Consequently, due to the transient nature of this injectable, it is possible that HA laryngoplasty may have impacted voice outcomes at earlier time points that were not captured in our study.

We chose HA due to its excellent safety profile, as it rarely elicits an inflammatory immune response [35]. We considered using calcium hydroxyapatite, which is known to last longer that HA. However, there have been several reports of adverse reactions when using this injectable for vocal cord augmentation, negatively impact voice outcomes [31]. There were no significant differences in RFS between the groups throughout our study, highlighting its safety for use in patients with laryngeal cancer. Furthermore, there were no adverse reactions or airway obstruction in the treatment group. All patients were discharged on their first post-operative day. Our laryngeal preservation rate in this study was 93.3%, which is on par with the literature.

It has been demonstrated that deeper and more extensive cordectomies (type III-V) are associated with worse voice outcomes [9]. Importantly, most patients in our cohort had a type IV or V cordectomy, which is associated with worse subjective voice outcomes post-operatively. Additionally, advanced age correlates with worse voice outcomes [26]. Although the average age in our cohort was 70.2 years; we found no difference in VHI-10 or MPT in patients under the age of 70 years in either group. Tumors involving the anterior commissure represent an aggressive subgroup of laryngeal SCC, with lower RFS and local control [7, 36]. Although anterior commissure resection is thought to result in worse voice outcomes, the direct impact on subjective and objective outcomes in unknown. There were no significant differences between the groups with respect to anterior commissure resection in our study.

Our study has several weaknesses. We report on a small sample size, and larger multi-centre studies are needed for more informed conclusions. We had fewer patients at later time points, increasing the likelihood of attrition bias. This occurred primarily as patients failed to return for follow-up, instead choosing to be seen by surgeons closer to home to avoid a longer commute. Data at these timepoints should therefore be interpreted with caution. Compared to awake procedures, it is difficult to assess the impact of injection laryngoplasty on vocal needs and/or dynamic glottic closure in patients under general anesthetic. This can undermine the amount of injectable delivered intra-operatively, therefore affecting the glottic gap and voice outcomes postoperatively. Although offered, none of our patients underwent post-operative voice therapy, as access to therapists specializing in voice is scarce. Therefore, its impact on voice outcomes in our cohort is not fully clarified. Ongoing smoking has been reported to negatively impact local disease control rates, as well as VHI-10 scores in early glottic cancers treated with radiation therapy [37]. This was not addressed in our trial, and should be accounted for in future studies investigating voice outcomes after TLM for glottic cancers. We only reported on MPT as an objective measure of voice outcomes. The impact of injection laryngoplasty on shimmer, jitter and harmonic-to-noise ratio following TLM remains elusive. Additionally, voice assessment tools such as the voice-vibratory assessment with laryngeal imaging (VALI), and the consensus auditory-perception evaluation of voice (CAPE-V) [38] could have been used as to assess voice outcomes. Future studies will focus on longer term follow-up for patients in our cohort to help elucidate voice and oncologic outcomes at later time points. Although our study focused on T1 and T2 lesions, much heterogeneity exists in the resultant surgical defects post-TLM. Additionally, owing to the subjectivity inherent in voice questionnaires, demonstrating a statistically significant difference between the groups is difficult. Future studies should focus on voice outcomes of injection laryngoplasty stratified by ELS cordectomy staging.

## Conclusions

Voice quality is an important outcome in the treatment of early glottic cancer. We report on a single-blinded, RCT investigating the impact of injection laryngoplasty on voice outcomes post-TLM for T1 and T2 lesions. No differences in MPT scores were seen between the groups throughout the study. Although VHI-10 scores improved over time amongst all patients in the study, no significant improvement was seen when comparing the treatment and control groups. Further studies are required to assess the impact of injection laryngoplasty on voice outcomes after TLM.

## Data Availability

All data is stored in a secured database within the hospital according to research ethics guidelines.

## Abbreviations

TLM: Transoral laser microsurgery
SCC: Squamous cell carcinoma
VHI-10: Voice Handicap Index-10
MPT: Maximum phonation time
RFS: Recurrence free survival
OS: Overall survival
DSS: Disease specific survival
ELS: European Laryngological Society
HA: Hyaluronic acid
RT: Radiation therapy
VALI: Voice-vibratory assessment with laryngeal imaging
CAPE-V: Consensus auditory-perception evaluation of voice
